# Association of serum cyclooxygenase-2 levels with hand-foot syndrome in patients receiving capecitabine: an exploratory analysis of D-TORCH study

**DOI:** 10.3332/ecancer.2025.1967

**Published:** 2025-08-15

**Authors:** Ghazal Tansir, Akhil Santhosh, Akash Kumar, Hemavathi Baskarane, Mohit Kumar Divakar, Vishakha Hooda, Arundhati J R Dev, Chandra Prakash Prasad, Ishaan Gupta, Saran Kumar, Pranay Tanwar, Atul Sharma, Sameer Bakhshi, Atul Batra

**Affiliations:** 1 Department of Medical Oncology, Dr. B.R.A. IRCH, All India Institute of Medical Sciences, New Delhi 110029, India; 2 Department of Medical Oncology, National Cancer Institute, All India Institute of Medical Sciences, Haryana 124105, India; 3 Department of Biochemical Engineering and Biotechnology, Indian Institute of Technology Delhi, New Delhi 110016, India; 4 Kusuma School of Biological Sciences, Indian Institute of Technology Delhi, New Delhi 110016, India; 5 Department of Laboratory Oncology, Dr. B.R.A. IRCH, All India Institute of Medical Sciences, New Delhi 110029, India; * Shared first authorship.

**Keywords:** hand-foot syndrome, cyclooxygenase, supportive care, biomarkers

## Abstract

**Background:**

The D-TORCH trial demonstrated superiority of 1% topical diclofenac over placebo in preventing capecitabine-induced hand-foot syndrome (HFS). We conducted an exploratory analysis of this study to assess the relationship between HFS and serum levels of the inflammatory marker, cyclooxygenase-2 (COX-2).

**Methods:**

Serum COX-2 levels were measured in patients in the D-TORCH study's experimental and placebo arms at baseline and 12 weeks of capecitabine-based therapy or at the development of HFS (whichever occurred earlier) and in 20 age-matched healthy controls using a human COX-2 ELISA kit (E-EL-H5574).

**Results:**

233 (88.5%) patients of the D-TORCH cohort (*n* = 263) underwent serial COX-2 analysis. The population was female predominant (*n* = 165, 70.8) with a median age of 47 years (range: 19–73), including breast (*n* = 130, 55.8%) and gastrointestinal cancers (*n* = 103, 44.2%). 31 (13.3%) patients developed any-grade HFS, with 25 (10.7%) having grade 2 or worse HFS. Mean serum COX-2 levels at baseline and 12 weeks did not show a statistically significant difference (mean + standard deviation, 3.41 + 2.15 ng/ml versus 3.35 + 2.40 ng/ml, p = 0.69); however, baseline levels in patients were significantly higher than healthy controls (*p* < 0.001). No statistically significant difference was found between serial COX-2 levels by gender, use of topical diclofenac, type of malignancy or severity of HFS.

**Conclusion:**

Serum COX-2 levels did not show a significant change with capecitabine-based therapy, regardless of the use of topical diclofenac possibly reflecting the predominant stromal production of the enzyme. This finding highlights the need to assess HFS-affected tissues for local COX-2 immuno-expression along with further blood-based biomarkers.

## Introduction

Capecitabine is an oral fluoropyrimidine with demonstrated efficacy in several cancers in the neoadjuvant, adjuvant and palliative settings [1–6]. Hand-foot syndrome (HFS) is a dose-limiting side effect that can affect over half the patients receiving capecitabine [7]. HFS manifests as palmoplantar numbness, tingling, erythema, edema, desquamation or ulceration of skin [8]. It is associated with pain, worsened quality of life, loss of fingerprint quality and superimposed infections [9].

Possible mechanisms of capecitabine-induced HFS include cyclooxygenase-2 (COX-2)-induced inflammation, accumulation of metabolites in palms and soles due to local concentration of thymidylate phosphorylase and genetic variants of ATP-binding cassette transporters [10,11]. COX-2 facilitates inflammation by metabolism of arachidonic acid into prostaglandin H, leading to the production of prostaglandin E2 [12]. Signs of inflammation have been demonstrated in HFS-affected tissues, such as white blood cell infiltration, vacuolar degeneration and vascular dilation, indicating the role of COX-2 in capecitabine-induced HFS [13, 14]. Thus, celecoxib was explored to prevent capecitabine-associated HFS in a randomised controlled trial demonstrating a significant reduction in the incidence of capecitabine-associated HFS among patients with colorectal cancer [7]. However, it is not routinely used due to concerns regarding cardiac and gastrointestinal adverse events.

We previously reported the D-TORCH study, a phase III randomised controlled trial that demonstrated superiority of topical 1% diclofenac gel versus placebo for preventing capecitabine-associated HFS. There was a significant reduction in Common Terminology Criteria for Adverse Events (CTCAEs) grade 1-3 (6.1% versus 18.1%) and grade 2 or higher HFS (3.8% versus 15%) with topical diclofenac compared to placebo [15, 16]. We present results of a planned assay of serum COX-2 at baseline and 12 weeks of therapy (or HFS, whichever was earlier) among patients of the D-TORCH study.

## Methods

### Study design

This investigator-initiated, phase III randomised, double-blind, placebo-controlled trial was conducted between January 2021 and February 2023 with its study protocol and primary results published earlier [15, 16]. It was prospectively registered with the Clinical Trials Registry of India (CTRI/202101/030592) and approved by the Institute Review Board (IECPG-82/27.01.2021).

Participants were randomised to receive topical 1% diclofenac or placebo for 12 weeks. The primary endpoint was grade 2 or higher HFS, and secondary endpoints included all-grade HFS, HFS-related dose changes of capecitabine and time to develop HFS.

### Sample collection and laboratory assay

Five millilitres of blood from participants was collected at baseline and 12 weeks of therapy or the development of HFS, whichever occurred earlier. Additionally, 5 ml of blood was drawn at a single point from 20 healthy controls.

Serum was analysed for COX-2 levels using a human COX-2 ELISA kit (Cat # E-EL-H5574, Elabscience Biotechnology Inc., Houston, TX, USA) following the sandwich ELISA method. Pre-coated micro ELISA plate wells with antibodies against COX-2 were utilised. Serum samples were added to the wells and incubated for 90 minutes to facilitate antigen binding, followed by adding a biotinylated detection antibody and avidin-conjugated horseradish peroxidase, with each step accompanied by incubation and several washings.

A substrate solution was introduced to trigger a colorimetric reaction, which resulted in a blue color change in wells containing Human COX2. The reaction was halted with a stop solution, producing a yellow color. Optical density (OD) was measured at 450 nm using a spectrophotometer. The OD values were directly proportional to the COX-2 concentration and determined by plotting a standard curve. The kit has a detection sensitivity of 0.19 (ng/ml) and a detection range of 0.31–20 ng/ml.

### Statistical analysis

Baseline clinical characteristics were presented using descriptive statistics, including median (inter-quartile range), mean, standard deviation (SD0 and frequencies. Paired samples *t*-test was used to compare mean (SD) COX-2 values at baseline and 12 weeks among the study cohort and various sub-groups. A *p*-value of < 0.05 was considered a priori to be statistically significant. Statistical analysis was done using GraphPad Prism version 10.3.1 for Windows, GraphPad Software, Boston, Massachusetts USA and SPSS version 29.0 (IBM Corp. Released 2023. IBM SPSS Statistics for Windows, Version 29.0.2.0 Armonk, NY: IBM Corp).

## Results

As published, the D-TORCH study randomised 264 patients to topical diclofenac gel (*n* = 131) and placebo (*n* = 133) arms between February 2021 and January 2023 with 263 patients analysed for study outcomes [15]. In this full cohort, grade 2–3 HFS was observed in 3.8% participants in the diclofenac group and 15.0% in the placebo group (95% confidence interval (CI), 4.3–18.1; *p* = 0.003). Grade 1–3 HFS was lower in the diclofenac than in the placebo group (6.1% versus 18.1%; 95% CI, 4.1–19.6).

Among 263 patients in the D-TORCH study, 233 (88.5%) patients underwent paired assessment of serum COX-2 levels at baseline and 12 weeks of treatment. The clinical profile of the patients included in the entire D-TORCH cohort and this particular analysis is described in Table 1. The biomarker-assessment population had a median age of 47 years (range: 19–73) and female predominance (*n* = 165, 70.8%). The predominant subtype of disease was breast cancer (*n* = 130, 55.8%) and most patients had metastatic disease (*n* = 119, 51.1%).

**Figure 1.**
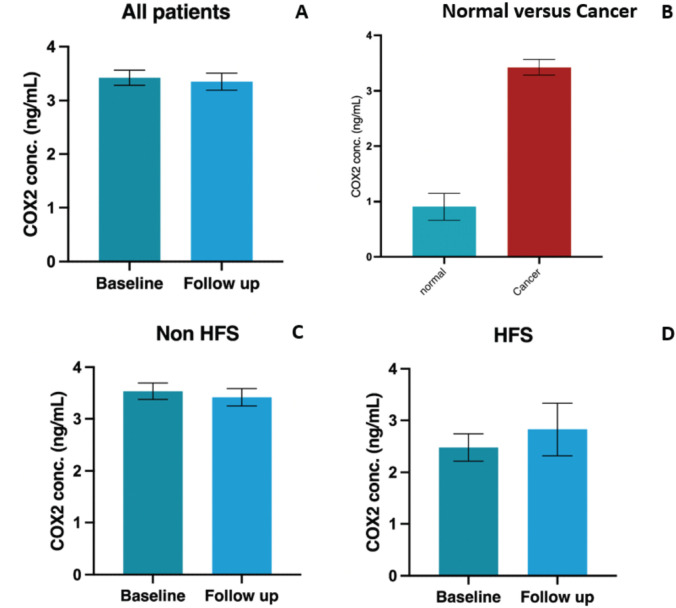
Graphical representation of COX-2 concentrations in populations (a): All patients included in the study cohort (b): Normal (healthy control) population and patients with malignancy (c): Baseline and 12-week levels in patients without HFS (d): Baseline and 12-week levels in patients with HFS. Abbreviations: COX-2: Cycloooxygenase-2, conc: concentration, ng/ml: nanogram per milliliter, HFS: Hand foot syndrome

31 (13.3%) patients in the biomarker-assessment population (*n* = 233) developed HFS with >/= grade 2 HFS present in 25 (10.7%). Of these HFS-affected patients (*n* = 31), 8 (25.8%) and 23 (74.2%) were in the topical diclofenac and placebo arms, respectively. Mean serum COX-2 levels at baseline and 12 weeks were 3.41 ng/ml (SD: 2.15, range: 0.18–13.9) and 3.35 ng/ml (SD: 2.40, range: 0.18–12.04), respectively (*p* = 0.69, SD: 2.31, 95% CI: -0.23-0.35). Mean serum COX-2 levels in healthy controls (0.93 ng/ml, SD: 0.97, range: 0–2.6), were statistically significantly lower compared to baseline COX-2 levels in the overall patient cohort (*p* < 0.001, SD: 2.02, 95% CI:1.02–3.36) (Figure 1).

### Subgroup analysis

Difference between mean COX-2 levels at baseline and 12 weeks was not statistically significantly different among subgroups by gender, treatment arms, occurrence of HFS, severity of HFS (CTCAE grade 0–1 and grade >/=2 HFS, other toxicities (CTCAE grade 1 and >/= grade 2), type of chemotherapy administered (capecitabine monotherapy versus combination), type of primary malignancy (breast versus GI cancers). The difference between the mean baseline and 12-week COX-2 in each subgroup with standard deviation and p values is presented in Table 2.

## Discussion

We present the biomarker analysis among participants of the D-TORCH trial, which found no significant difference in serum COX-2 levels within 12 weeks of therapy. Limited studies have explored biomarkers for capecitabine-induced HFS, including the association of serum and red blood cell (RBC) folate levels with grade 2 or higher HFS [17]. Higher serum and RBC folate levels were found to be significantly correlated with the occurrence of grade 2 or worse HFS on multivariate analysis. However, only 1 in 150 individuals had true folate deficiency, reflected by levels of 283 nmol/l. Hence, true folate deficiency was present in only a small fraction of patients. It was, thus, opined by the authors that larger studies needed to be conducted to study the folate levels among different populations on capecitabine. It was also suggested that lower dietary folates in East Asian diets could prevent capecitabine-associated HFS by providing less substrate for the conversion of fluorouracil metabolites to thymidylate synthase. Serum dihydropyrimidine dehydrogenase levels were also found to be associated with grade 1 and 2 HFS among patients on celecoxib for prevention of capecitabine-induced HFS [7]. There are currently no available studies on the utility or cost-effectiveness of the use of these biomarkers in predicting the development of HFS, and this knowledge will be beneficial in choosing the appropriate test for further use. Currently, these tests are available for commercial use, but the cost can be a deterrent for routine use in resource-limited settings among those at risk of HFS.

High COX-2 immunoexpression was described in multiple myeloma [18], oropharyngeal cancers [19, 20] and malignant melanoma [21], also serving as an adverse prognostic factor in melanoma [22]. These studies utilised tissue COX-2 expression because the enzyme is predominantly secreted near the tumour stroma [23]. However, there is no literature assessing serum COX-2 and its association with outcomes or adverse events in cancer despite the enzyme being a part of systemic inflammation. Its role as a biomarker for treatment-related adverse events remains under-explored. Due to capecitabine-induced HFS being a potential inflammatory side effect and COX-2 inhibitors exhibiting benefit in its prevention, prospective serum COX-2 analysis was part of the D-TORCH study.

Baseline COX-2 levels in patients with cancer were significantly higher than controls, indicating the validity of the testing apparatus and the possibility of higher tumour-related inflammation in the patients. COX-2 levels were not significantly altered after 12 weeks of treatment with capecitabine, regardless of HFS and use of topical diclofenac. This persistent elevation could indicate active inflammation secondary to causes such as residual tumour and the use of chemotherapy. The use of topical diclofenac and the resulting reduction in HFS did not translate into a decrease in COX-2 levels, possibly due to the local rather than systemic action of the experimental drug.

Our study did not include tissue COX-2 assessment, due to which inferences on COX-2 expression in HFS are incomplete. Despite this limitation, ours is the first study to explore a potential biomarker of HFS in a commonly used drug. Future studies on HFS could be designed to test COX-2 immunoexpression on the palms or soles with paired serum assays. Other blood-based inflammatory biomarkers, such as prostaglandin E2 may also be assayed. We highlight the need for assaying tissue and blood-based inflammatory markers, including COX-2, that could serve as biomarkers to identify patients at high risk of developing capecitabine-related HFS.

## Conclusion

Among participants of the D-TORCH study, delta serum COX-2 levels were not significantly different by treatment arms or occurrence of HFS, highlighting the need to study tissue expression of COX-2 as a biomarker for capecitabine-induced HFS.

### Statements and declarations

The study has been cleared by the Institute Review Board (IECPG-82/27.01.2021) and performed in accordance with the ethical standards laid down in the 1964 Declaration of Helsinki and its later amendments. The trial was registered prospectively under the Clinical Trials Registry of India (CTRI/2021/01/030592 (D-TORCH).

The persons included in the study gave their informed consent prior to inclusion.

The authors declare that they do not have any conflict of interest.

## Funding

Supported in part by Alkem Laboratories Ltd and Indian Association of Supportive Care in Cancer.

## Author contributions

All authors contributed to the study conception and design. Material preparation, data collection and analysis were performed by Ghazal Tansir and Akhil Santhosh. The first draft of the manuscript was written by Ghazal Tansir and all authors commented on previous versions of the manuscript. All authors read and approved the final manuscript.

## Figures and Tables

**Table 1.** Clinical and demographic details of full study cohort and patients who underwent paired COX-2 level assay.

Characteristics	Full study cohort (*n* = 263)		COX-2 assay cohort (*n* = 233)	
Median age (years, range),	47 (19–73)		47 (19–73)	
Sex distribution Female (*n*, %) Male (*n*, %)	187 (71.1) 76 (28.9)		165 (70.8) 68 (29.2)	
Type of chemotherapy (*n*, %) Monotherapy Combination	108 (41.1) 155 (58.9)		95 (40.8) 138 (59.2)	
Type of malignancy (*n*, %) Breast GI	148 (56.3) 115 (43.7)		130 (55.8) 103 (44.2)	
Prior chemotherapy (*n*, %) Received Not received	98 (37.3) 165 (62.7)		86 (36.9) 147 (63.1)	
Stage of malignancy (*n*, %) II III IV Not known	4 (1.5) 126 (47.9) 132 (50.2) 1 (0.4)		4 (1.7) 109 (46.8) 119 (51.1) 0 (0)	
HFS (*n*, %) Grade I Grade II Grade III	Diclofenac arm (*n* = 130) 3 (2.3) 2 (1.5) 3 (2.3)	Placebo arm (*n* = 133) 4 (3.0) 13 (9.8) 7 (5.3)	Diclofenac arm (*n* = 118) 3 (2.5) 2 (1.7) 3 (2.5)	Placebo arm (*n* = 115) 3 (2.6) 13 (11.3) 7 (6)
Abbreviations: COX-2: Cycloooxygenase-2; GI: Gastrointestinal; HFS: Hand Foot Syndrome				

**Table 2.** COX-2 levels in various subgroups of the study cohort.

S.No.	Subgroup (*n*)	Mean COX-2 levels: Week 12 minus baseline (SE mean)	Standard deviation	*P*-value (95% CI)
1.	a. Topical diclofenac (118) b. Placebo (115)	−0.23 (0.20) 0.11 (0.22)	2.24 2.40	0.26 (-0.64-0.17) 0.60 (-0.32-0.56)
2.	a. Male (68) b. Female (165)	−0.22 (0.21) −0.009 (0.18)	2.43 2.42	0.29 (-0.65-0.19) 0.95 (-0.38-0.36)
3.	a. Capecitabine monotherapy (95) b. Combination (138)	−0.43 (0.24) −0.07 (0.19)	2.43 2.26	0.86 (-0.53-0.45) 0.71 (-0.45-0.31)
4.	a. Breast cancer (130) b. GI cancer (103)	−0.22 (0.21) 0.14 (0.21)	2.43 2.18	0.29 (-0.65-0.19) 0.49 (-0.27-0.57)
5.	a. Without HFS (202) b. With HFS (31)	−0.99 (0.16) 0.19 (0.34)	2.39 1.90	0.55 (-0.43-0.23) 0.56 (-0.50-0.89)
6.	a. Grade 0-1 HFS (208) b. >/=2 Grade 2 HFS (25)	−0.10 (0.16) 0.35 (0.40)	2.36 2.01	0.50 (-0.43-0.21) 0.19 (-0.48-1.18)
7.	Capecitabine-associated diarrhea: a. Grade 0–1 (180) b. >/=Grade 2 (53)	−0.008 (0.16) −0.233 (0.37)	2.2 2.72	0.95 (-0.33-0.31) 0.53 (-0.98-0.57)
8.	Capecitabine-associated mucositis a. Grade 0–1 (176) b. >/=Grade 2 (57)	−0.26 (0.16) −0.16 (0.36)	2.19 2.72	0.87 (-0.35-0.30) 0.65 (-0.88-0.55)
9.	Capecitabine-associated myelosuppression a. Grade 0-1 (226) b. >/=Grade 2 (7)	−0.38 (0.15) −0.73 (0.93)	2.32 2.47	0.80 (-0.34-0.26) 0.46 (-3.02-1.55)
Abbreviations: CI: Confidence interval, SE: Standard error, HFS: Hand-foot syndrome, GI: gastrointestinal				
